# Supplementary material for: Epigenetic modifications regulate cultivar-specific root development and metabolic adaptation to nitrogen availability in wheat
Source: Nat Commun. 2023 Dec 12;14:8238. doi: 10.1038/s41467-023-44003-6 (PMC10716289; doi:10.1038/s41467-023-44003-6)
Supplement: Supplementary file 1 — Supplementary Information [file 41467_2023_44003_MOESM1_ESM.pdf]

# Epigenetic modifications regulate cultivar-specific root development and metabolic adaptation to nitrogen availability in wheat

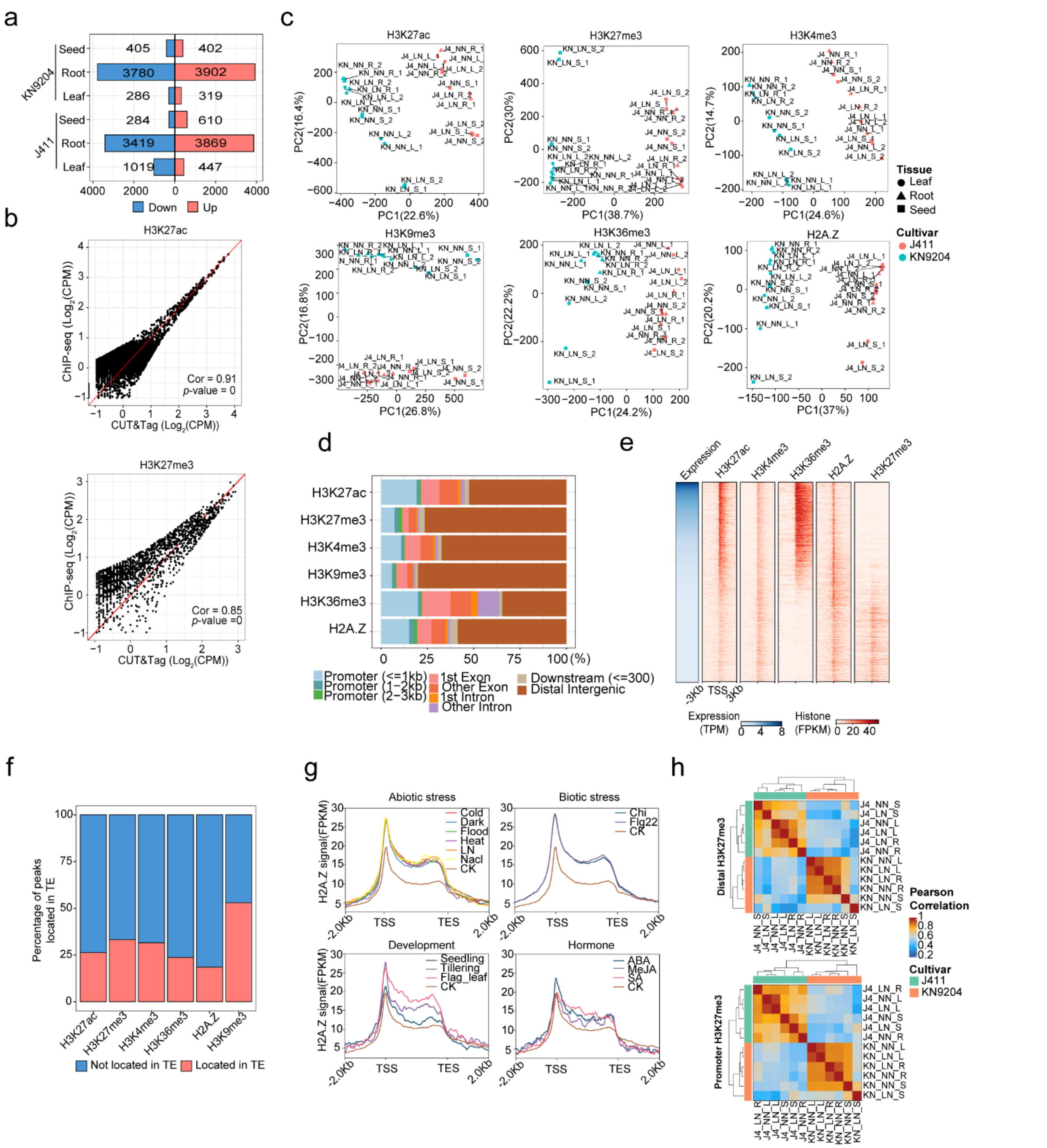

**Supplementary Fig.1. NUE epigenome dataset**

a. The number of DEGs in the tissues in response to LN in KN9204 and J411
b. Correlation of ChIP-seq and CUT&Tag of H3K27ac[20] and H3K27me3[21] in wheat.
c. PCA plots of H3K27ac, H3K27me3, H3K4me3, H3K9me3, H3K36me3, and H2A.Z in the NUE epigenome dataset. Each dot represents one sample. Two biological replicates were sequenced for each stage. Abbreviations were as follow: KN, KN9204; J4, J411; NN, normal nitrogen; LN, low nitrogen; S, seed; R, root; L, flag leaf.
d. Peak distributions in gene regions of different histone marks in the wheat genome.
e. Heatmaps of epigenetic marks for all annotated wheat genes which were sorted according to their expression levels.
f. Proportion of peaks located in TE regions for the six different histone marks.
g. H2A.Z profiles of DEGs that respond to external stimuli (abiotic and biotic stress), RNA-seq data from[1].
h. Cross-correlation heatmaps of all H3K27me3 peaks which are located in distal or promoter regions separately. Abbreviations: KN, KN9204; J4, J411; NN, normal nitrogen; LN, low nitrogen; S, seed; R, root; L, flag leaf.

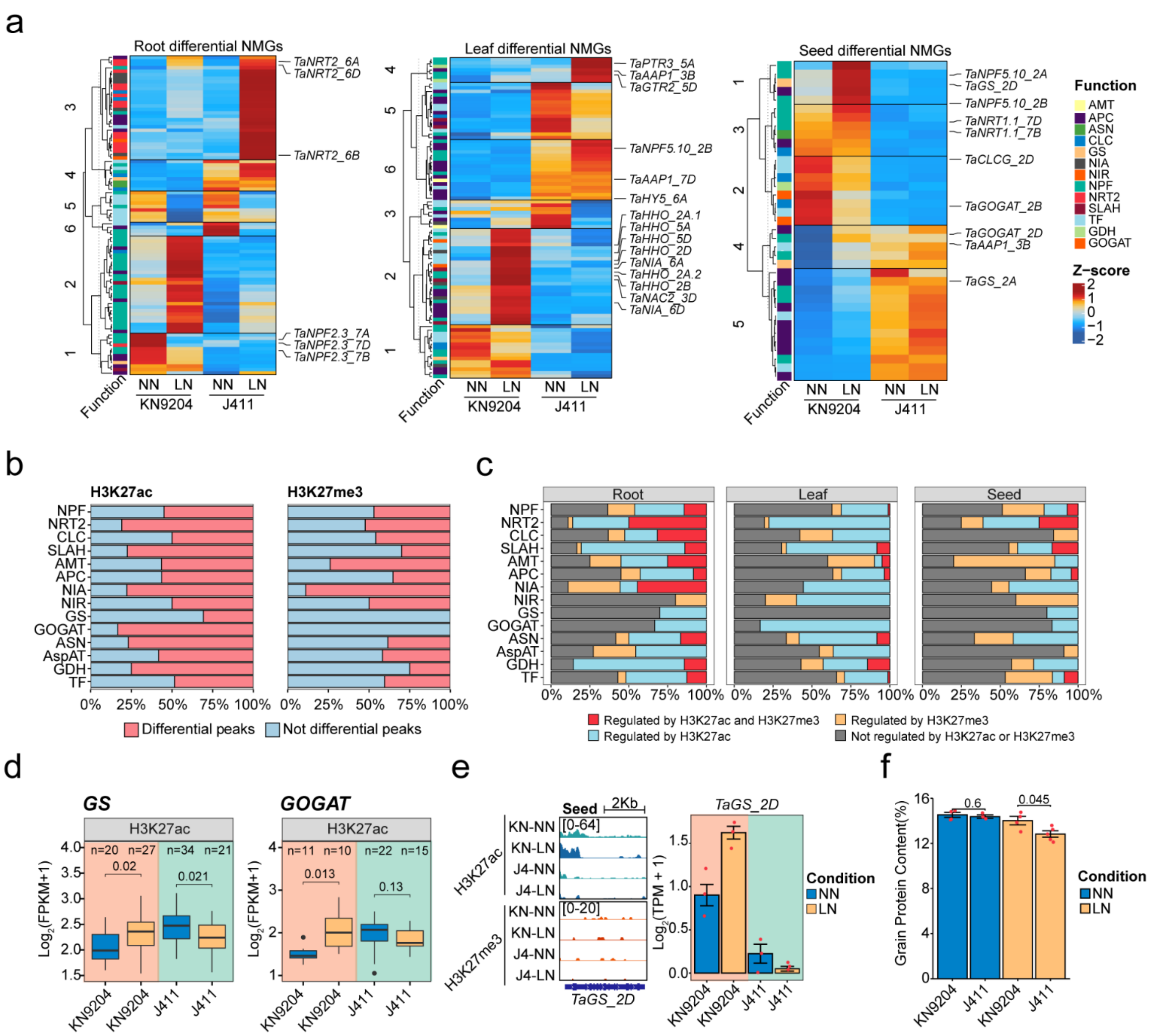


**Supplementary Fig.2. Dynamic changes in the transcriptomes and epigenomes of NMGs**

a. K-means clustering of LN-induced differentially-expressed NMGs in roots, leaves, and seeds of KN9204 and J411. See also Supplemental Table 1. Abbreviations: NPF, NRT1/PTR FAMILY; NRT2, Nitrate transporter 2; CLC, Chloride channel protein; SLAH, Slow anion channel-associated homologues; AMT, Ammonium transporter; APC, The amino acid–polyamine–choline transporter superfamily; NIA, Nitrate reductase; NIR, Nitrite reductase; GS, Glutamine synthetase; GOGAT, Glutamate synthetase; ASN, Asparagine synthetase; AspAT, Aspartate aminotransferase; GDH, Glutamate dehydrogenase; TF, transcription factor.

b. Percentage of NMGs regulated by differential H3K27ac and H3K27me3 in different categories. Abbreviations were same with (a).

c. Percentage of NMGs regulated by H3K27ac, H3K27me3, or combined effect. Abbreviations were same with (a).

d. H3K27ac levels of *GS* and *GOGAT* in the seeds of KN9204 and J411 between the two nitrogen availability levels (two-sided Wilcox test). Different shades indicate KN9204 and J411 separately. Boxplots show the median, third and first quartiles. The numbers indicate the number of peaks used in the analysis.

e. Representative tracks showing histone modifications and transcriptional changes of *TaGS_2D* in seeds for the two wheat cultivars and two nitrogen levels. Different shades indicate KN9204 and J411 separately. Expression data shown as mean ± s.d. of 3 biological replicates.

f. Grain protein content (GPC) in seeds of KN9204 and J411 under different nitrogen conditions (two-sided Student's t-test). Data shown as mean ± s.d. of 4/5 biological replicates.

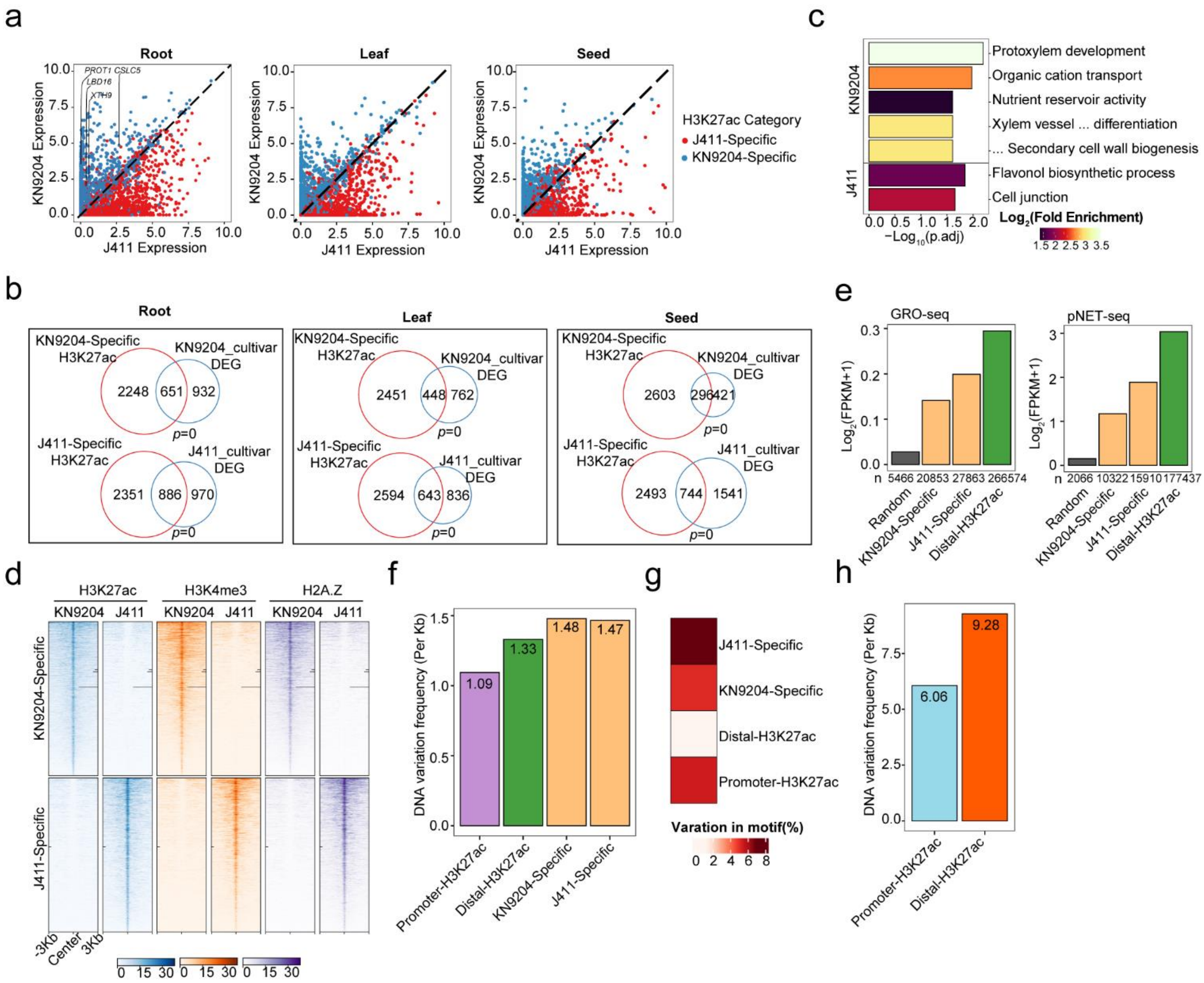


**Supplementary Fig.3. The characteristic of cultivar-biased H3K27ac peaks**

a. Mean gene expression in J411 (x-axis) versus mean gene expression in KN9204 (y-axis) for genes associated with proximal cultivar-specific H3K27ac peaks separately in three tissues.
b. Overlap between genes marked by cultivar-specific H3K27ac (red circle) and DEGs between cultivars (blue circle) in three tissues. Significance assessed using one-sided Fisher's exact test for the overlaps.
c. GO enrichment of genes that are marked by proximal cultivar-specific H3K27ac peaks in KN9204 and J411 (two-sided Fisher's exact test, BH for multiple comparisons).
d. Heatmaps showing the co-localization between cultivar-specific H3K27ac peaks and H3K4me3/H2A.Z.
e. The mean expression level of eRNA in cultivar-specific and promoter/distal H3K27ac peaks, random regions in genome was selected as control. The numbers indicate the number of peaks used in the analysis.
f. The DNA variation frequency (Per Kb) between KN9204 and J411 located in cultivar-specific and promoter/distal H3K27ac peaks.
g. The DNA variation frequency (Per Kb) between KN9204 and J411 located in TF binding motif of cultivar-specific and promoter/distal H3K27ac peaks.
h. The DNA variation frequency (Per Kb) located in promoter/distal H3K27ac peaks from wheat resequencing[30,31].

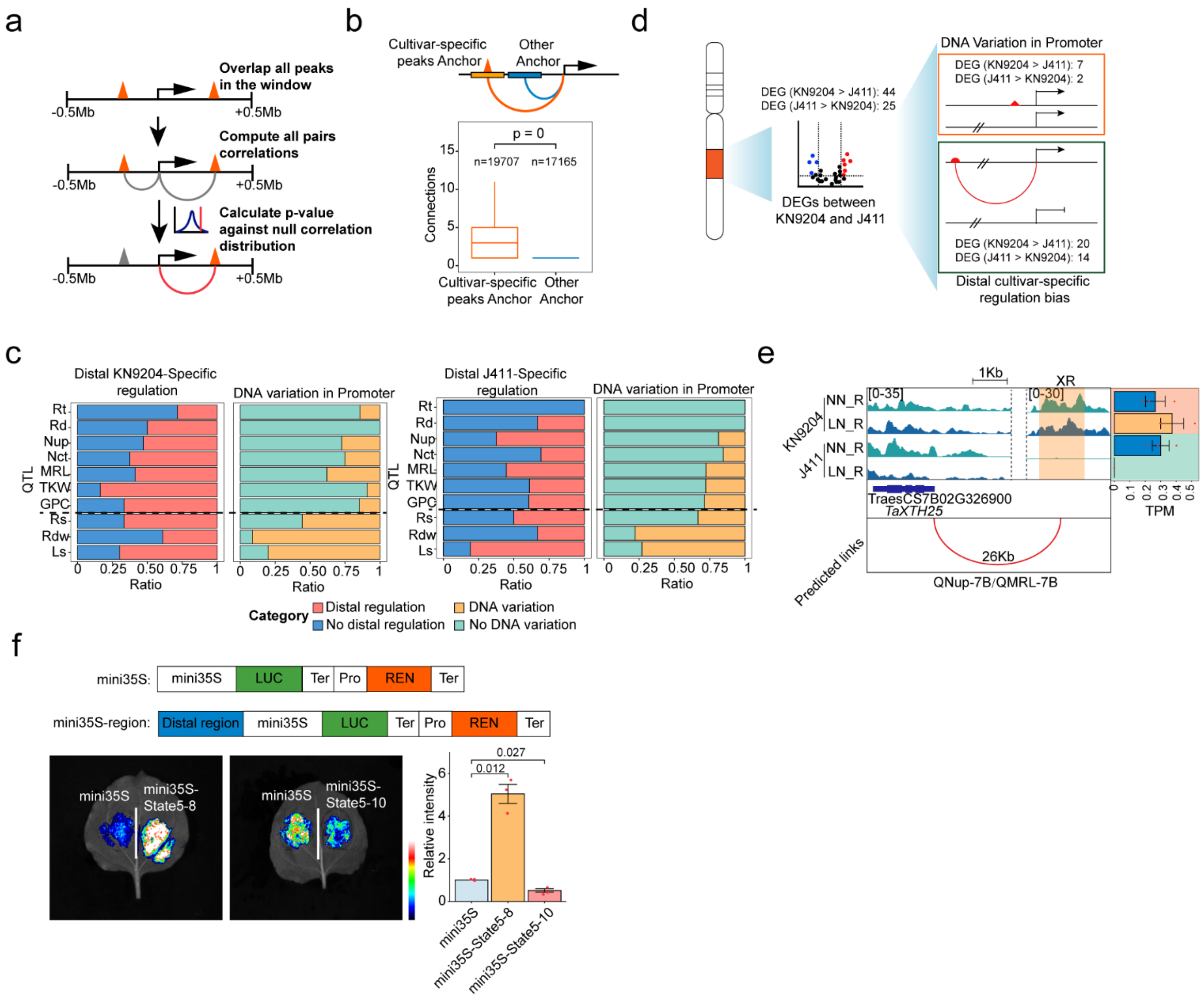

**Supplementary Fig.4. The functional influence of distal cultivar-biased H3K27ac peaks**

a. Schematic diagram showing the approach used to link distal cultivar-specific H3K27ac peaks to genes.

b. Cross validation of distal cultivar-specific H3K27ac peaks assigned by Hi-C data[2] (two-sided Wilcox test). Boxplots show the median, third and first quartiles. The numbers indicate the number of peaks used in the analysis.

c. Fraction of DEGs with distal KN9204-specific/J411-specific regulation or DNA variation within the promoters located in QTLs between KN9204 and J411. Abbreviations: Rt: root tip number; Rd: root diameter; Nup: Nitrogen uptake content; Nct: Nitrogen concentration; MRL: Maximum root length; TKW: Thousand-kernel weight; GPC: Grain protein content; Rs: Root surface area; Rdw: Root dry weight; Ls: flag leaf size.

d. Schematic diagram illustrating the distal cultivar-specific regulation and DNA variation in promoter for DEGs located in the qMRL-7B.

e. Representative tracks of *TaXTH25* regulated by distal cultivar-specific H3K27ac peaks in *QMRL-7B*. The distal regulatory regions of *TaXTH25* was denoted as "XR". Abbreviations: NN_R: NN_Root, LN_R: LN_Root. Expression data shown as mean ± s.d. of 3 biological replicates.

f. Positive and negative control of luciferase reporter assay, region tested (positive: State5-8, negative: State5-10) got from data published before[41] (two-sided Student's t-test). Data shown as mean ± s.d. of 3 biological replicates.

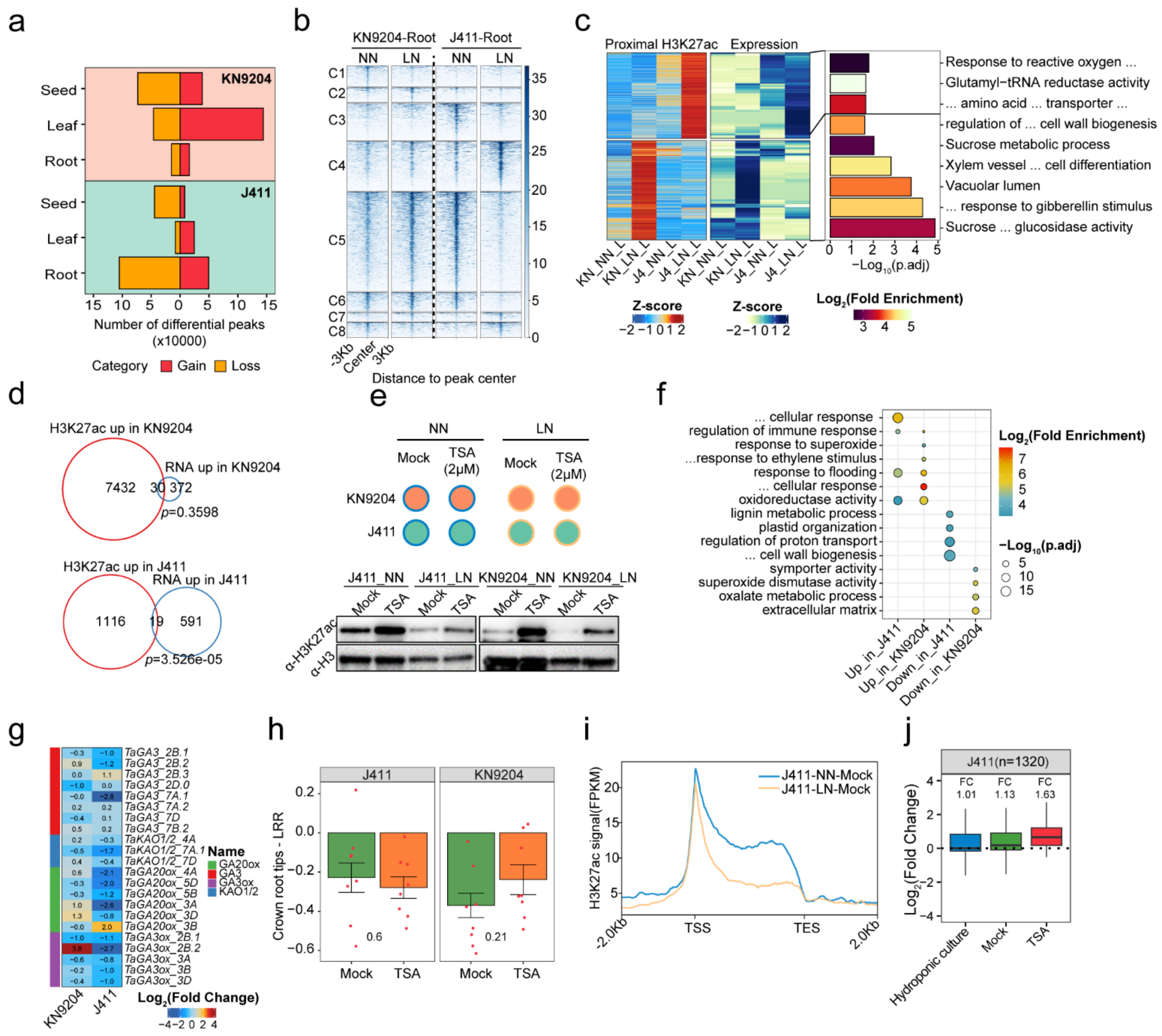


**Supplementary Fig.5. Dynamic H3K27ac changes in KN9204 and J411**

a. The number of differential H3K27ac peaks in response to LN of three tissues (seeds, roots, leaves) in KN9204 and J411.

b. Heatmaps showing the differential H3K27ac peaks in the root of KN9204 and J411 under LN and NN conditions.

c. Dynamic H3K27ac, corresponding expression changes and GO enrichments in the flag leaves of KN9204 and J411 (two-sided Fisher's exact test, BH for multiple comparisons). Abbreviations: KN_NN_L: KN9204_NN_Leaf, KN_LN_L: KN9204_LN_Leaf, J4_NN_L: J411_NN_Leaf, J4_LN_L: J411_LN_Leaf.

d. Overlap between genes with up-regulated H3K27ac and the up-regulated DEGs for KN9204 and J411 in the seeds. Significance assessed using one-sided Fisher's exact test for the overlaps.

e. Experiment design and western blotting of TSA (2 μM) treatment of KN9204 and J411 seedlings under the two nitrogen conditions. Abbreviations: KN_NN_R: KN9204_NN_Root, KN_LN_R: KN9204_LN_Root, J4_NN_R: J411_NN_Root, J4_LN_R: J411_LN_Root. Experiment repeated 2 times with similar results.

f. GO enrichment of genes that was differential expressed upon TSA treatment under NN condition (two-sided Fisher's exact test, BH for multiple comparisons).

g. The transcription fold change of GA biosynthesis gene (mentioned in the TSA treatment of *Populus trichocarpa*[3]) upon TSA treatment.

h. The LN-response-ratio (LRR) of crown root tips for KN9204 and J411 (two-sided Student's *t*-test). Data shown as mean ± s.d. of 8 biological replicates.

i. H3K27ac profiles of genes (in Fig. 4e) under different nitrogen conditions in J411.

j. The transcription fold change of genes (in Fig. 4e) in response to LN under hydroponic culture, mock, and TSA treatment. Boxplots show the median, third and first quartiles. The numbers indicate the number of genes used in the analysis.

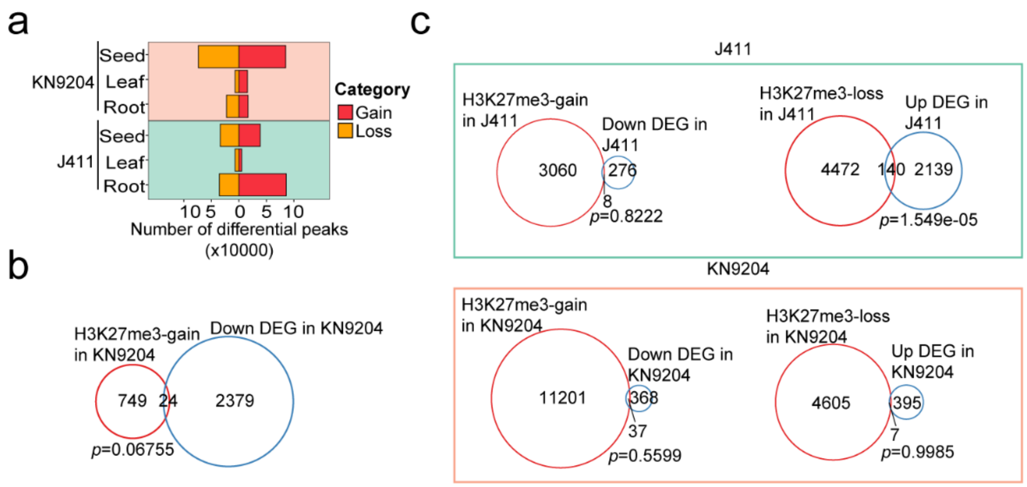


**Supplementary Fig.6. Dynamic H3K27me3 changes in KN9204 and J411**

a. The number of differential (gain or loss) H3K27me3 peaks in response to LN of three tissues in KN9204 and J411.

b. Overlap between genes with LN-induced H3K27me3 and the down-regulated DEGs in the roots of KN9204. Significance assessed using one-sided Fisher's exact test for the overlaps.

c. Overlap between genes LN-induced H3K27me3 and the down-regulated DEGs in the seeds of J411 and KN9204. Significance assessed using one-sided Fisher's exact test for the overlaps

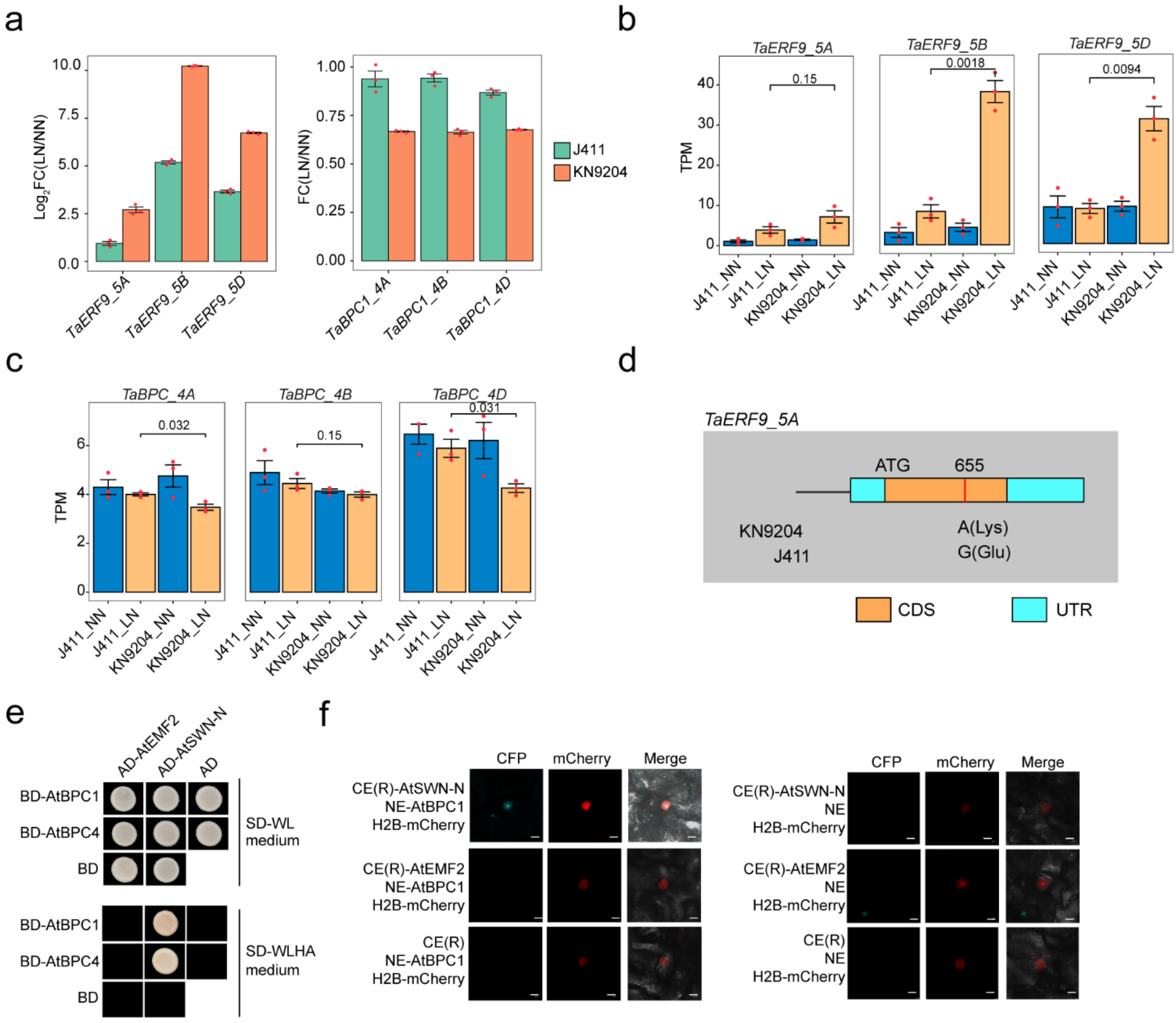


**Supplementary Fig.7. Recruitment of H3K27me3 under LN condition in KN9204 and J411**

a. The transcription fold change of *ERF9* and *BPC1* under LN condition in KN9204 and J411. Expression data shown as mean ± s.d. of 3 biological replicates.

b. The expression of *ERF9* under different nitrogen conditions in KN9204 and J411. Expression data shown as mean ± s.d. of 3 biological replicates (two-sided Student's *t*-test).

c. The expression of *BPC1* under different nitrogen conditions in KN9204 and J411. Expression data shown as mean ± s.d. of 3 biological replicates (two-sided Student's *t*-test).

d. Schematic diagram illustrating DNA variation in *ERF9_5A* between KN9204 and J411.

e. Yeast two-hybrid (Y2H) assays showing the interaction between AtBPC1 and PRC2 components AtEMF2, AtSWN-N (N-terminal of AtSWN). Transformed yeast cells were cultured on synthetic media lacking Leu and Trp (SD-WL) or Leu, Trp, His, and Ade (SD-WLHA).

f. Bimolecular fluorescence complementation (BiFC) analysis displaying the interaction between AtBPC1 and AtSWN-N, AtEMF2. H2B-mCherry was used as control for transformation and localization of nuclei. Scale bars = 10 mm. CE(R), C terminal (right) of eYFP; NE, N terminal of eYFP. Experiment repeated 2 times with similar results.

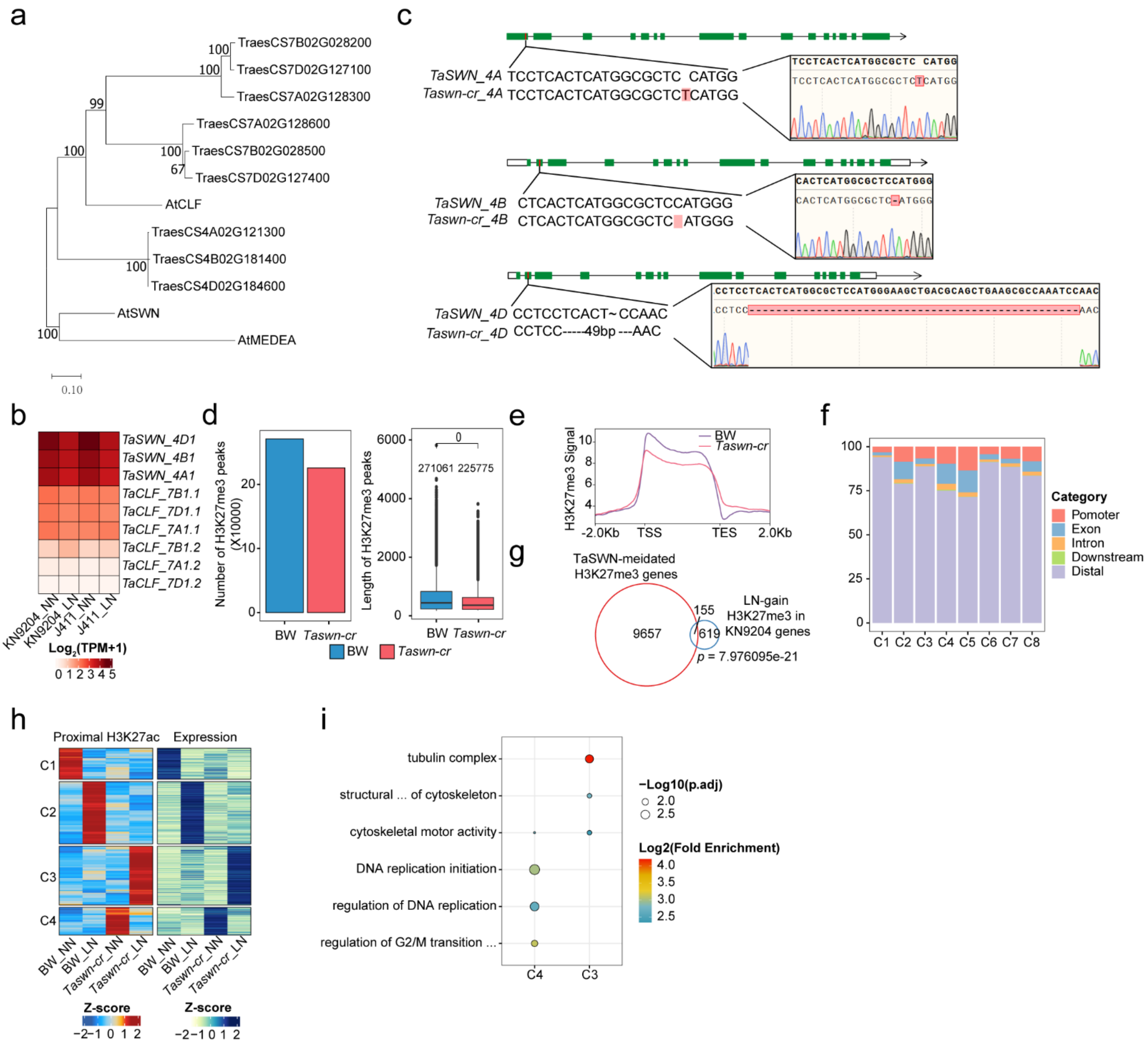

**Supplementary Fig.8. The generation and influence to global H3K27me3 level of *Taswn-cr***

a. A phylogenetic tree showing the evolutionary relationships between Ez proteins (one part of PRC2) from *Arabidopsis* and wheat.

b. Expression of *TaSWN* and *TaCLF* genes under LN and NN conditions in KN9204 and J411.

c. DNA sequence identification the mutated target sites in the three *TaSWN* genes in the *Taswn-cr* mutant.

d. Peak numbers and lengths of H3K27me3 peaks in *Taswn-cr* and BW plants under different nitrogen conditions (two-sided Wilcox test). Boxplots show the median, third and first quartiles. The numbers indicate the number of peaks used in the analysis.

e. Profiles of H3K27me3 levels in *Taswn-cr* and BW under normal nitrogen conditions.

f. Peak distribution of the differential H3K27me3 peaks in Fig. 6a.

g. Overlap between genes marked by TaSWN-dependent H3K27me3 and genes marked by gain-H3K27me3 under LN condition in KN9204. Significance assessed using one-sided Fisher's exact test for the overlaps.

h. The dynamic H3K27ac and transcription change in BW and *Taswn-cr*.

i. GO enrichment of the different cluster genes from (h) (two-sided Fisher's exact test, BH for multiple comparisons).

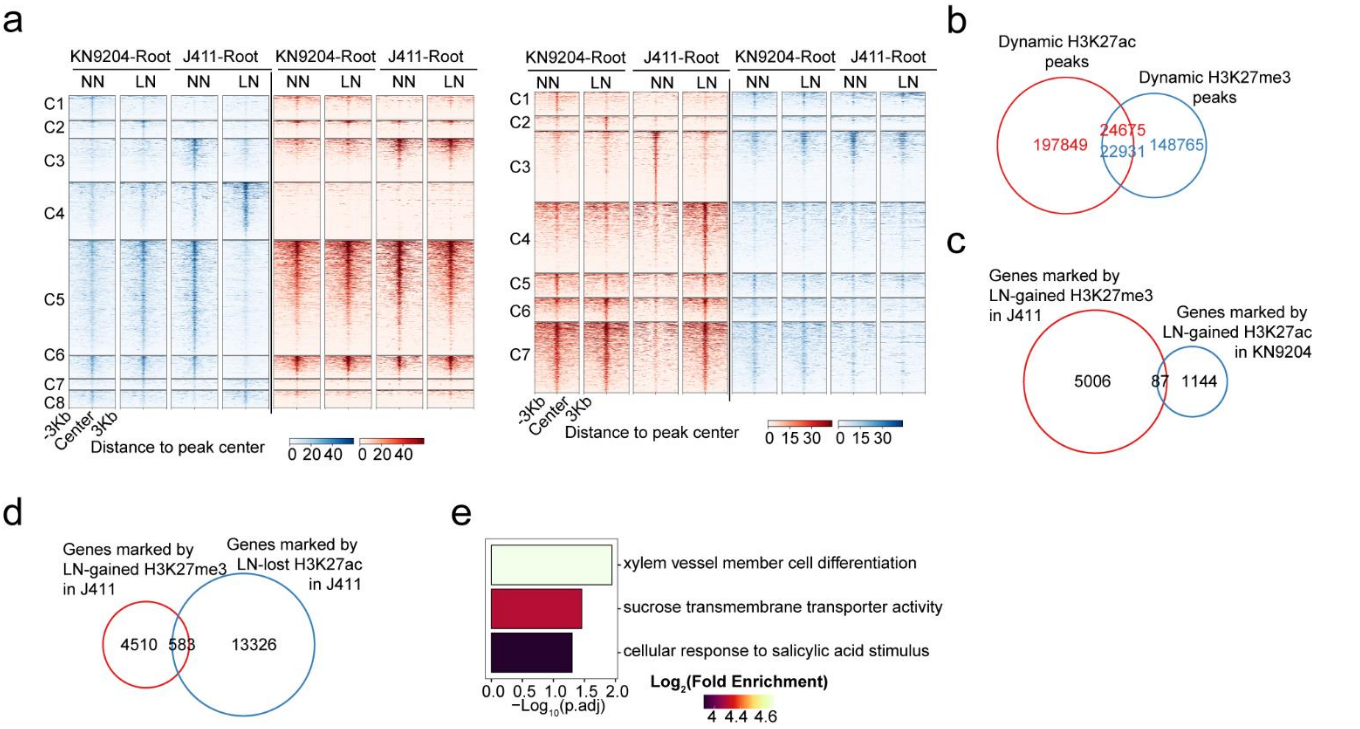


**Supplementary Fig.9. Overlap between dynamic H3K27ac and H3K27me3**

a. Heatmap of dynamic regions showing H3K27ac and H3K27me3 change pattern together, the H3K27me3 change in dynamic H3K27ac regions (left panel) the H3K27ac change in dynamic H3K27me3 regions (right panel).
b. The overlap of dynamic H3K27ac and H3K27me3 peaks.
c. The overlap of genes regulated by dynamic H3K27ac and H3K27me3 peaks related to root development (LN-induced H3K27ac in KN9204, LN-induced H3K27me3 peaks in J411).
d. The overlap of genes regulated by dynamic H3K27ac and H3K27me3 peaks related to root development (LN-lost H3K27ac in J411, LN-induced H3K27me3 peaks in J411).
e. The GO enrichment of genes overlapped in (d) (two-sided Fisher's exact test, BH for multiple comparisons).

## References


1 Wang, M. et al. An atlas of wheat epigenetic regulatory elements reveals subgenome divergence in the regulation of development and stress responses. Plant Cell 33, 865-881 (2021). https://doi.org:10.1093/plcell/koab028

2 Concia, L. et al. Wheat chromatin architecture is organized in genome territories and transcription factories. Genome Biol 21, 104 (2020). https://doi.org:10.1186/s13059-020-01998-1

3 Ma, X., Zhang, C., Zhang, B., Yang, C. & Li, S. Identification of genes regulated by histone acetylation during root development in Populus trichocarpa. BMC Genomics 17, 96 (2016). https://doi.org:10.1186/s12864-016-2407-x
