# Supplementary material for: Epigenetic modifications regulate cultivar-specific root development and metabolic adaptation to nitrogen availability in wheat
Source: Nat Commun. 2023 Dec 12;14:8238. doi: 10.1038/s41467-023-44003-6 (PMC10716289; doi:10.1038/s41467-023-44003-6)
Supplement: Supplementary file 3 — Description of Additional Supplementary Files [file 41467_2023_44003_MOESM3_ESM.pdf]

Description of Additional Supplementary Files

Supplementary Data 1: Differentially expressed NMGs between KN9204 and J411.

Supplementary Data 2: Differential H3K27ac peaks of NMG between KN9204 and J411.

Supplementary Data 3: Differential H3K27me3 peaks of NMG between KN9204 and J411.

Supplementary Data 4: Assignment of distal cultivar-specific H3K27ac regions.

Supplementary Data 5: Distal regulations of DEGs in QMRL-7B.

Supplementary Data 6: ERFs identified in QTL.

Supplementary Data 7: Primers used in this study.
