# Supplementary material for: Epigenetic modifications regulate cultivar-specific root development and metabolic adaptation to nitrogen availability in wheat
Source: Nat Commun. 2023 Dec 12;14:8238. doi: 10.1038/s41467-023-44003-6 (PMC10716289; doi:10.1038/s41467-023-44003-6)
Supplement: Supplementary file 11 — Reporting Summary [file 41467_2023_44003_MOESM11_ESM.pdf]

nature portfolio

Corresponding author(s): Jun Xiao

Last updated by author(s): Nov 24, 2023

Reporting Summary

Nature Portfolio wishes to improve the reproducibility of the work that we publish. This form provides structure for consistency and transparency in reporting. For further information on Nature Portfolio policies, see our Editorial Policies and the Editorial Policy Checklist.

Statistics

For all statistical analyses, confirm that the following items are present in the figure legend, table legend, main text, or Methods section.

n/a	Confirmed	
☐	☒	The exact sample size (*n*) for each experimental group/condition, given as a discrete number and unit of measurement
☐	☒	A statement on whether measurements were taken from distinct samples or whether the same sample was measured repeatedly
☐	☒	The statistical test(s) used AND whether they are one- or two-sided<br>*Only common tests should be described solely by name; describe more complex techniques in the Methods section.*
☒	☐	A description of all covariates tested
☐	☒	A description of any assumptions or corrections, such as tests of normality and adjustment for multiple comparisons
☐	☒	A full description of the statistical parameters including central tendency (e.g. means) or other basic estimates (e.g. regression coefficient) AND variation (e.g. standard deviation) or associated estimates of uncertainty (e.g. confidence intervals)
☐	☒	For null hypothesis testing, the test statistic (e.g. *F*, *t*, *r*) with confidence intervals, effect sizes, degrees of freedom and *P* value noted<br>*Give P values as exact values whenever suitable.*
☒	☐	For Bayesian analysis, information on the choice of priors and Markov chain Monte Carlo settings
☒	☐	For hierarchical and complex designs, identification of the appropriate level for tests and full reporting of outcomes
☐	☒	Estimates of effect sizes (e.g. Cohen's *d*, Pearson's *r*), indicating how they were calculated

Our web collection on statistics for biologists contains articles on many of the points above.

Software and code

Policy information about availability of computer code

Data collection

CUT&Tag were performed using the Illumina NovaSeq 6000 platform. Luciferase activities were measured by GLOMAX 20/20. Root architecture was scanned by ScanMaker i8000 plus. Luciferase images were taken by Tanon Imaging System. 15N-content was detected using an isotope ratio mass spectrometer (Isoprime 100)

Data analysis

Code used in this study was deposited in the GitHub (https://github.com/ZhangHao-995/NUE-pipeline). Raw reads were cleaned by fastp (v0.20.1) with "--detect_adapter_for_pe" parameter. Clean reads of RNA-seq libraries were aligned to wheat reference genome (IWGSC RefSeq v1.1 using hisat2 (v2.1.0). Clean reads of CUT&Tag libraries were aligned to wheat reference genome (IWGSC RefSeq v1.1) using bwa mem algorithm (v 0.7.17) with "-M" parameter. Aligned reads of CUT&Tag were filtered by samtools (v1.5) with "-F 1,804 -f 2 -q 30" parameter and deduplicated by Picard (v 2.20.5-0). The peak calling was done by macs2 (v 2.1.4). For narrow peaks (H3K27ac, H3K4me3, and H2A.Z) and broad peaks (H3K27me3, H3K36me3, and H3K9me3), parameters "-p 1e-3 --keep-dup all -g 14600000000" and "--keep-dup all -g 14600000000 --broad --broad-cutoff 0.05" were used. Peak was annotated to the wheat genome using the ChIPseeker (v1.30.3). featureCounts (v2.0.1) was used to count the number of paired mapping reads that overlap each annotated gene (IWGSC Annotation v1.1) with the parameter "-p -P -B -C". deeptools (v3.3.0) was used to convert bam files into bigwig files and visualization for epigenomic data. IGV (v2.8.0.01) was used to visualize the track of epigenomic data. MAnorm (v1.3.0) was used for the quantitative comparison of CUT&Tag signals between samples with the following criteria: |M value| > 1 and $P < 0.05$. Fimo (v4.11.2) was used to scan motifs. GO annotation files were generated from IWGSC Annotation v1.1 and clusterProfiler (v4.2.2) was used for enrichment analysis. chromHMM (v1.21) was used. "BinarizeBam" and "LearnModel" commands with default parameters were used for chromatin-state (CS) annotation. R (https://cran.r-project.org/;version 4.2.1) was used to compute statistics

For manuscripts utilizing custom algorithms or software that are central to the research but not yet described in published literature, software must be made available to editors and reviewers. We strongly encourage code deposition in a community repository (e.g. GitHub). See the Nature Portfolio guidelines for submitting code & software for further information.

Data

Policy information about availability of data

All manuscripts must include a data availability statement. This statement should provide the following information, where applicable:

- Accession codes, unique identifiers, or web links for publicly available datasets
- A description of any restrictions on data availability
- For clinical datasets or third party data, please ensure that the statement adheres to our policy

The raw sequence data were deposited in the Genome Sequence Archive (https://bigd.big.ac.cn/gsa) under accession number CRA009936 (https://bigd.big.ac.cn/gsa/browse/CRA009936); the transcriptome used was under BioProject accession numbers CRA003969 (https://bigd.big.ac.cn/gsa/browse/CRA003969). Source data are provided with this paper.

Research involving human participants, their data, or biological material

Policy information about studies with human participants or human data. See also policy information about sex, gender (identity/presentation), and sexual orientation and race, ethnicity and racism.

Reporting on sex and gender	not applicable
Reporting on race, ethnicity, or other socially relevant groupings	not applicable
Population characteristics	not applicable
Recruitment	not applicable
Ethics oversight	not applicable

Note that full information on the approval of the study protocol must also be provided in the manuscript.

Field-specific reporting

Please select the one below that is the best fit for your research. If you are not sure, read the appropriate sections before making your selection.

☒ Life sciences ☐ Behavioural & social sciences ☐ Ecological, evolutionary & environmental sciences

For a reference copy of the document with all sections, see nature.com/documents/nr-reporting-summary-flat.pdf

Life sciences study design

All studies must disclose on these points even when the disclosure is negative.

Sample size	The sample size and the results of statistical analyses are described in the relevant figures or method section. Sample size was determined based on experimental trials to allow for confident statistical analyses.
Data exclusions	No data that pass quality control were exclusion from analysis.
Replication	CUT&Tag have two biological replications. Other experiments involving measurements and quantifications were repeated at least two times using biological materials produced independently. All replication attempts were successful.
Randomization	Plants were grown side by side to minimize unexpected environmental variations during growth and experimentation.
Blinding	Blinding was not applied due to lack of prior knowledge and expectation of the final results.

Reporting for specific materials, systems and methods

We require information from authors about some types of materials, experimental systems and methods used in many studies. Here, indicate whether each material, system or method listed is relevant to your study. If you are not sure if a list item applies to your research, read the appropriate section before selecting a response.

Materials & experimental systems

n/a	Involved in the study
☐	☒ Antibodies
☒	☐ Eukaryotic cell lines
☒	☐ Palaeontology and archaeology
☒	☐ Animals and other organisms
☒	☐ Clinical data
☒	☐ Dual use research of concern
☐	☒ Plants

Methods

n/a	Involved in the study
☒	☐ ChIP-seq
☒	☐ Flow cytometry
☒	☐ MRI-based neuroimaging

Antibodies

Antibodies used

Anti-H3K27ac antibody (Abcam, Cat#ab4729) with 1/50 dilution, Anti-H3K27me3 antibody (CST, Cat#C36B11) with 1/50 dilution, Anti-H3K4me3 antibody (Abcam, Cat#ab8580) with 1/50 dilution, Anti-H3K9me3 antibody (Abcam, Cat#ab8898) with 1/50 dilution, Anti-H3K36me3 antibody (Abcam, Cat#ab9050) with 1/50 dilution, Anti-H2A.Z antibody (Gift from Prof. Roger Deal lab, the same as "https://doi.org/10.1371/journal.pgen.1008326") with 1/50 dilution.

Validation

All primary antibodies are commercially available. All validation statements can be found on the manufacturers'websites (Antibodies-Online: https://www.antibodies-online.com/antibody; Abcam, https://www.abcam.com/nav/primary-antibodies) The following validation have been extracted from the manufacturer's webpage. The anti-H3K27ac antibody specifically recognizes the Histone H3-acetyl K27 modification and is expected to demonstrate broad species cross-reactivity due to high sequence conservation. The anti-H3K27me3 antibody specifically recognizes the Histone H3-tri methyl K27 modification and is expected to demonstrate broad species cross-reactivity due to high sequence conservation. The anti-H3K4me3 antibody specifically recognizes the Histone H3 tri methy-K4 modification and is expected to demonstrate broad species cross-reactivity due to high sequence conservation. The anti-H3K9me3 antibody specifically recognizes the Histone H3 tri methy-K4 modification and is expected to demonstrate broad species cross-reactivity due to high sequence conservation. The anti-H3K36me3 antibody specifically recognizes the Histone H3 tri methy-K4 modification and is expected to demonstrate broad species cross-reactivity due to high sequence conservation. Additionally, these antibody were validated in wheat in a previous study: Zhao, L., Yang, Y., Chen, J. et al. Dynamic chromatin regulatory programs during embryogenesis of hexaploid wheat. Genome Biol 24, 7 (2023).

Dual use research of concern

Policy information about dual use research of concern

Hazards

Could the accidental, deliberate or reckless misuse of agents or technologies generated in the work, or the application of information presented in the manuscript, pose a threat to:

No	Yes
☒	☐ Public health
☒	☐ National security
☒	☐ Crops and/or livestock
☒	☐ Ecosystems
☒	☐ Any other significant area

Experiments of concern

Does the work involve any of these experiments of concern:

No	Yes
☒	☐ Demonstrate how to render a vaccine ineffective
☒	☐ Confer resistance to therapeutically useful antibiotics or antiviral agents
☒	☐ Enhance the virulence of a pathogen or render a nonpathogen virulent
☒	☐ Increase transmissibility of a pathogen
☒	☐ Alter the host range of a pathogen
☒	☐ Enable evasion of diagnostic/detection modalities
☒	☐ Enable the weaponization of a biological agent or toxin
☒	☐ Any other potentially harmful combination of experiments and agents
